# Supplementary material for: Content-rich biological network constructed by mining PubMed abstracts
Source: BMC Bioinformatics. 2004 Oct 8;5:147. doi: 10.1186/1471-2105-5-147 (PMC528731; doi:10.1186/1471-2105-5-147)
Supplement: Additional File 2 — The original results of the above study (non-essential files are deleted to keep the file size under the limit set by BMC bioinformatics). [file 1471-2105-5-147-S2.bz2 › chilibotAdditionalFile2/dip20/148ID9160881E524/html/CBL_LYN.html]

 


 **CBL** and **LYN** 
  
Found 24 abstracts in PubMed, retrieved 20.  
 

 What does Google say? 
 PDF only 
| .edu only 

---

**Interactive relationship** (e.g. stimulation, inhibition, etc)

**Inhibitory relationship**- v  **Cbl**  suppresses apoptosis and differentiation, possibly through enhancement of  **Lyn** , Syk, and PI3 kinase activities and Bcl 2.  Ref: 11378270 Exp Hematol, 2001
- In hck fgr  **lyn**  triple mutant cells, which are defective in spreading on fibronectin coated surfaces in vitro and show impaired migration in vivo,  **Cbl**  tyrosine phosphorylation is blocked,  **Cbl**  protein levels are low, adhesion dependent translocation of  **Cbl**  to the membrane is impaired and  **Cbl**  associated, membrane localized phosphatidylinositol 3 PI 3 kinase activity is dramatically reduced.  Ref: 9687507 EMBO J, 1998
- This phosphorylation was intact in Btk and  **Cbl**  deficient DT40 cells, but was drastically suppressed in  **Lyn** , Syk, or BCAP deficient DT40 cells.  Ref: 12627965 Biochemistry, 2003
**Neutral relationship**- When yeast were transformed with catalytic  **Lyn** , an interaction between p85 s SH2 domain and  **Cbl**  occurred.  Ref: 9160881 Oncogene, 1997
- Using yeast two hybrid analysis, we found that c  **Cbl**  directly couples  **Lyn**  to PI 3 kinase.  Ref: 12149655 Oncogene, 2002
- **Lyn**  associates with and phosphorylates a small set of molecules, including c  **Cbl** .  Ref: 12149655 Oncogene, 2002
- This process has been proposed to be mediated by an interaction between the Src family kinase  **LYN**  and the p85 subunit of PI3K and or through p85 membrane recruitment mediated by  **CBL**  and or CD19.  Ref: 10551821 J Biol Chem, 1999
- Through its Src homology 3 SH3 and SH2 domains, the Src kinase  **Lyn**  interacts with a small number of phosphoproteins, such as Shc,  **Cbl** , and Vav, which regulate cell cycle and the cytoskeleton.  Ref: 12456510 Blood, 2003
- Following ligation of surface integrins by fibronectin, the p120 c  **cbl**   **Cbl**  protein rapidly becomes tyrosine phosphorylated and associated with the Src family kinases Fgr and  **Lyn** .  Ref: 9687507 EMBO J, 1998
- Aggregation of FcgammaRIIa, the low affinity receptor for monomeric IgG, activates nonreceptor protein tyrosine kinases such as  **Lyn** , Hck, and Syk, potentially driving the phosphorylation of the downstream adaptor proteins, including  **Cbl**  and or Nck.  Ref: 9851874 Exp Cell Res, 1998
- Binding studies indicated that  **Cbl**  could bind to glutathione S transferase fusion proteins encoding the unique, Src homology domain 3, and SH2 domains of Fyn, Hck, or  **Lyn** .  Ref: 9890970 J Biol Chem, 1999
- Tyrosine phosphorylation of Vav and c  **Cbl**  was restored in common by a  **Lyn** , a Hck, and a Src, but Fc gamma RIIB tyrosine phosphorylation, which is implicated in negative signaling, was reconstituted solely by a  **Lyn**  and a Hck.  Ref: 10861086 J Immunol, 2000
- From the data, we propose the following three step process of PI 3 kinase activation complexes of  **Lyn**   **Cbl**  and  **Cbl**  p85 exist without ligand stimulation, upon ligand binding,  **Lyn**  becomes active and phosphorylates  **Cbl** , and  **Cbl**  s tyrosine phosphorylated residue serves as a docking site for the SH2 domains of p85 thereby stabilizing the complex and activating PI 3 kinase.  Ref: 9160881 Oncogene, 1997
- Basal activities of  **Lyn** , Syk, and PI3 kinase were elevated in the v  **Cbl**  line.  Ref: 11378270 Exp Hematol, 2001

**Non-interactive relationship** (e.g. studied together, co-existance, homology, etc.)

- Involvement of Shc and  **Cbl**  PI 3 kinase in  **Lyn**  dependent proliferative signaling pathways for G CSF.  Ref: 10644984 Oncogene, 2000
- Using the yeast two hybrid screen, we demonstrate an in vivo association of  **Lyn**  s SH3 and SH2 domains with the proline rich domain of  **Cbl** .  Ref: 9160881 Oncogene, 1997
- G CSF induced the tyrosine phosphorylation of  **Cbl**  and increased activity of PI 3 kinase in wild type and syk deficient, but non in  **lyn**  deficient, cells.  Ref: 10644984 Oncogene, 2000
- In this study, we demonstrate that HGF induces strong tyrosine phosphorylation of the proto oncogene product c  **Cbl**  in B cells and increases  **Cbl**  association with the Src family tyrosine kinases Fyn and  **Lyn** , as well as with phosphatidylinositol 3 kinase and CrkL.  Ref: 12244174 J Immunol, 2002
- Thus, the  **Lyn** ,  **Cbl**  PI 3 kinase, and Shc non Ras dependent pathways correlate with the ability of cells to respond to G CSF with increased DNA synthesis.  Ref: 10644984 Oncogene, 2000
- Enhanced activation of fyn and  **lyn**  kinases and the tyrosine phosphorylation of  **cbl**  were also observed.  Ref: 11157475 Blood, 2001
- Under our conditions, hydrogen peroxide induced PI3K and Akt activation was independent of  **Lyn** , Syk,  **Cbl** , BCAP, or Ras when each was eliminated individually either by mutation or by a specific inhibitor.  Ref: 12627965 Biochemistry, 2003
- In retinoic acid differentiated HL 60 cells, cross linking of FcgammaRs resulted in a marked increase in the tyrosine phosphorylation of FcgammaRIIa, p58  **lyn** , and p120 c  **cbl** , which was inhibited by a specific inhibitor of Src family protein tyrosine kinases.  Ref: 11698455 J Immunol, 2001
